# Culturally-adapted and audio-technology assisted HIV/AIDS awareness and education program in rural Nigeria: a cohort study

**DOI:** 10.1186/1472-698X-10-2

**Published:** 2010-02-02

**Authors:** Ighovwerha Ofotokun, Jose Nilo G Binongo, Eli S Rosenberg, Michael Kane, Rick Ifland, Jeffrey L Lennox, Kirk A Easley

**Affiliations:** 1 Department of Medicine, Division of Infectious Diseases, Emory University, Atlanta GA, USA; 2 Department of Mathematics & Statistics, Kennesaw State University, Kennesaw, GA, USA; 3 Department of Biostatistics & Bioinformatics, Rollins School of Public Health, Emory University, Atlanta GA, USA; 4 Voice for Humanity, 841 Corporate Drive, Suite 205, Lexington KY, USA

## Abstract

**Background:**

HIV-awareness programs tailored toward the needs of rural communities are needed. We sought to quantify change in HIV knowledge in three rural Nigerian villages following an integrated culturally adapted and technology assisted educational intervention.

**Methods:**

A prospective 14-week cohort study was designed to compare short-term changes in HIV knowledge between seminar-based education program and a novel program, which capitalized on the rural culture of small-group oral learning and was delivered by portable digital-audio technology.

**Results:**

Participants were mostly Moslem (99%), male (53.5%), with no formal education (55%). Baseline HIV knowledge was low (<80% correct answers for 9 of the 10 questions). Knowledge gain was higher (p < 0.0001 for 8 of 10 questions) in the integrated culturally adapted and technology-facilitated (n = 511) compared with the seminar-based (n = 474) program.

**Conclusions:**

Baseline HIV-awareness was low. Culturally adapted, technology-assisted HIV education program is a feasible cost-effective method of raising HIV awareness among low-literacy rural communities.

## Background

The acquired immunodeficiency syndrome (AIDS) epidemic continues to be a major challenge to global health and socio-economic development in many regions of the world [1-3]. An estimated 33 million people worldwide were living with HIV by the end of 2007; 2.7 million became newly infected and 2 million died from AIDS during that year [4]. In Nigeria, the estimated number of adults (>15 years) living with HIV in 2007 was 2.4 million [5,6]. Effective education programs that take into consideration the information needs of the predominantly rural inhabitants are needed to improve HIV prevention knowledge. In this report, we present the findings of a 14-week longitudinal evaluation of a culturally-adapted, small audio-technology HIV awareness campaign focused upon an oral message, compared with a conventional seminars/pamphlet based program in three rural communities in the northern Nigerian State of Kano.

## Methods

### Target Communities

Three villages (Miamakawa, Gaya South, and Kutama) were recruited in consultation with local organizations (Federation of Moslem Women Association of Nigeria (FOMWAN) and the Evangelical Church of West Africa (ECWA) with an extensive grass-roots presence in Northern Nigeria. These villages were small, with each inhabited by less than 30,000 people, and were relatively remote -- about a two-hour drive on unfinished roads from the nearest major city, Kano City. Miamakawa and Gaya South were assigned together as the intervention group due to their close proximity to one another. Kutama was designated as the control village. Kutama was geographically separated from the intervention villages, helping to minimize the information crossover during the evaluation period.

### HIV Education Content

HIV education materials, based on existing local HIV training curriculum, were modified to address basic knowledge about the virus, transmission risk as applicable to the community, practical risk reduction approaches, as well as local myth and misinformation that fuel the stigmatization of people living with HIV. To mirror the known cultural learning habits of these villages, the content was formatted into short stories, proverbs, parables, dramas, and folk songs in the indigenous Hausa language using characters and voices of recognized community role models (e.g., the local radio newscasters, assistants in the community place of worship, etc.). This information was arranged into a 3-hour audio program and loaded onto the specially-designed digital audio player described below. A one-page version of the content was also printed as pamphlets written in Hausa for distribution to the control community.

### Digital Audio Technology

Developed by Voice For Humanity (VFH), Lexington Kentucky, U.S.A., the digital audio device resembles an MP3-player and uses voice compression technology (125X) that allows up to five hundred hours of information to be stored in a removable microchip. The units are portable, fitting into a shirt pocket, and can be powered by solar-charged batteries or by wall-outlet supplied electricity. They have no moving parts and are resistant to wind, dirt and sand. Furthermore, the microchips can be easily replaced, allowing the audio devices to be used for other health and social development programs. The greater flexibility in program format and on-demand replay afforded by this technology gives it an advantage over radio in deploying media interventions.

### Intervention (HIV Awareness and Education Campaign)

The education program was conducted in the last two weeks of November 2005. In the intervention communities, trusted individuals recommended by the community chiefs (*Megeri*) were recruited as group leaders who would train others to play the digital devices in small listening groups. These groups included 5-10 people. After listening to the audio program, the groups were encouraged to discuss the contents. Participating villagers who expressed interest in helping with the campaign were further trained by the leaders and were provided with devices to continue in a similar manner, organizing new small learning groups. Approximately 3000 devices were disseminated in the intervention villages.

In the control village, the HIV education program mirrored previous HIV campaigns in the communities in order to allow the comparison of the new approach to the existing standard for that locality. The control HIV education program included a one-time open house seminar conducted in the local Hausa language by the ECWA HIV chief educator at the community rendezvous. The event consisted of a 60 minute HIV awareness lecture, followed by an all-day interactive question-and-answer session between villagers and project staff. To ensure a wider reach, the seminar was supplemented by the distribution of approximately 3000 of the aforementioned pamphlets to households in the community by trained volunteers. These volunteers also read the pamphlets aloud to recipients who requested help. The topics discussed at the seminar and the content of the pamphlet were similar to the HIV information distributed in the intervention community.

### Project Evaluation

**Figure 1:**
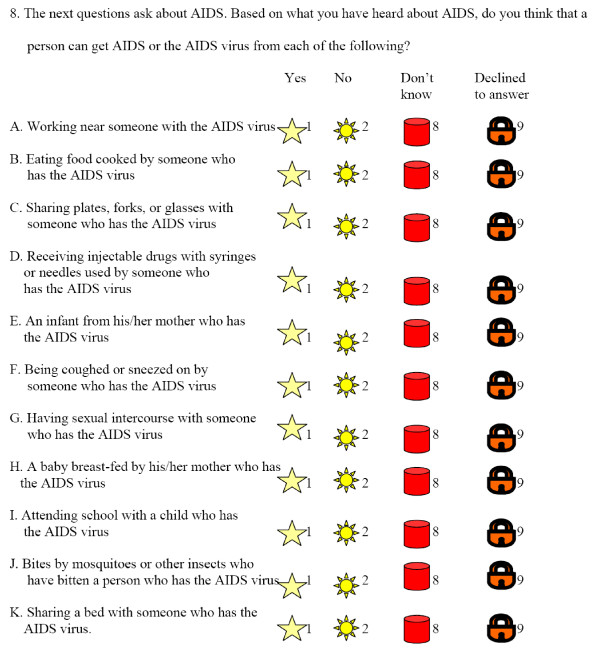
**Excerpt from the assessment tool showing the HIV knowledge questions of Table 2, in English translation**.

The assessment tool, which included questions designed to gauge the respondents' level of HIV awareness as well as attitudes and beliefs, was adapted from the AIDSCAP/WHO Voluntary HIV Counseling and Testing Efficacy Instrument. This instrument has been used in multiple studies including studies in three developing countries: Kenya, Tanzania, and Trinidad [7-9]. Because the target population was predominantly non-literate, questions were translated into Hausa and programmed for replay into the same type of digital audio devices used in the study intervention. The data collection instrument consisted of answer choices represented by recognizable and familiar objects in the communities rather than alphabetical characters (Figure 1).

Emory University personnel trained the 20 local field workers to administer the questionnaire using the audio device. Each field worker was assigned to districts within the target communities and instructed to go from house to house and identify individuals in his or her district who were willing to participate in the 14-week study. A local guide was paired with each field worker to ensure accurate tracking of participants during the evaluation period. As local Moslem culture restricted interactions between unrelated male and female individuals, male field workers were paired with female guides and vice-versa to ensure each team had access to both male and female participants.

Villagers were eligible to participate in the assessment if they were > 12 years of age, agreed to take part in the HIV campaign by listening to the audio device (intervention community) or to attend the HIV seminar or read the HIV education pamphlet given and/or read to them (control village). Other eligibility criteria included a willingness to participate and stay in the program for the entire duration, and having no more than a sixth grade education.

Participants responded to questions as they were played by the audio player by selecting the picture object on their answer sheet which in their view appropriately answered the question. This approach allowed for privacy in response to questions with little or no help from the field workers.

At week 14, the degree of community exposure in the intervention communities was assessed through a convenience sampling of individuals to assess whether they participated in the program or not. The reach of the seminar and pamphlet distribution was not assessed in the control village.

All participants provided oral informed consent before completion of the baseline assessment. This study was designed according to the ethical guidelines for human studies and approved by the Emory University Institutional Review Board and the ECWA Healthcare System Ethics Committee.

### Statistical Methods

Demographic data and responses to HIV knowledge questions were summarized and compared between different subgroups of the population. Chi-square tests were applied to examine differences between subgroups for the ten HIV knowledge questions. Multivariable logistic regression was used to determine whether baseline knowledge differences between the control and intervention groups remained after adjusting for gender and education. The primary outcome for this cohort study was change in response over time for a set of ten HIV knowledge questions. The outcome response was a correct or incorrect answer to each HIV question. Analyses for repeated measures were needed to properly account for the correlation between multiple observations from the same participant. Two approaches were used to analyze the data. First, McNemar's test of correlated proportions was used to test within group change by assessing symmetrical change among those participants in which changes in response occurred. Were learning not truly occurring, one would expect these changes to occur at random and the off-diagonal counts would be approximately equivalent. This test was performed separately for the control and intervention group to determine within-group change (baseline to week 7 and baseline to week 14). Differences in change between the control and intervention group were tested with the Pearson chi-square test for independence [10]. This test focused on whether the change in HIV knowledge between baseline and 7 weeks (and separately, baseline to 14 weeks) was related to the intervention. Following these univariate comparisons described above, we utilized Generalized Estimating Equations [GEE] modeling methods [11], which allow for covariate adjustment. The repeated response data at baseline, 7 weeks and 14 weeks were compared between intervention groups by performing a GEE logistic regression analysis implemented using SAS Proc Genmod using an exchangeable correlation structure for the repeated binary responses within participant for each of the HIV knowledge questions. These models were refit to adjust for gender and education. The model-based estimates are unbiased with unbalanced and missing data, so long as the missing data are non-informative (missing completely at random). All statistical tests were two-sided. A Bonferonni adjusted α of 0.005 (.05/10 questions) was used to assess differences in change between education programs for the HIV knowledge questions. The same adjustment for multiplicity was used when testing for within-group change at each follow-up visit for each of the HIV knowledge questions. For testing the covariates of education and gender in our GEE models, α = 0.05 was used.

## Results

Demographic characteristics of participants are summarized in Table 1. Most of the 1205 participants were Moslem (99%), married (80%), and between 15-45 years of age (78%). Approximately half of the cohort was female (47%).

**Table 1:** Demographic characteristics among 1205 participants in three rural Nigerian communities

	Overall		Control		Intervention	
	(n = 1205)		(n = 594)		(n = 611)	
	***%***	***Total***	***%***	***Total***	***%***	***Total***
Sex						
Male	53.5	(643/1201)	47.3	(280/592)	59.6	(363/609)
Female	46.5	(558/1201)	52.7	(312/592)	40.4	(246/609)
Age						
12 - 15 years	7.8	(93/1188)	7.3	(43/587)	8.3	(50/601)
15 - 45 years	77.9	(926/1188)	77.2	(453/587)	78.7	(473/601)
> 45 years	14.2	(169/1188)	15.5	(91/587)	13.0	(78/601)
Education^1^						
None	55.2	(651/1179)	61.4	(358/583)	49.2	(293/596)
Grades 1 - 3	6.3	(74/1179)	7.4	(43/583)	5.2	(31/596)
Grades 4 and 5	4.5	(53/1179)	3.8	(22/583)	5.2	(31/596)
Grade 6	34.0	(401/1179)	27.4	(160/583)	40.4	(241/596)
Marital Status^1^						
Married, monogamous	59.1	(656/1110)	69.5	(386/555)	48.6	(270/555)
Married, polygamous	20.9	(232/1110)	11.5	(64/555)	30.3	(168/555)
Separated	2.1	(23/1110)	0.5	(3/555)	3.6	(20/555)
Divorced	1.3	(14/1110)	0.5	(3/555)	2.0	(11/555)
Widowed	2.1	(23/1110)	1.3	(7/555)	2.9	(16/555)
Other/Missing	14.6	(162/1110)	16.6	(92/555)	12.6	(70/555)
Technology in the home^2^						
Radio	83.0	(1000/1205)	83.5	(496/594)	82.5	(504/611)
Telephone	4.6	(55/1205)	4.2	(25/594)	4.9	(30/611)
Television	13.4	(161/1205)	13.5	(80/594)	13.3	(81/611)
Information Sources^2^						
Newspaper	2.0	(24/1205)	1.5	(9/594)	2.5	(15/611)
Television	8.6	(104/1205)	11.8	(70/594)	5.6	(34/611)
Radio	84.4	(1017/1205)	86.2	(512/594)	82.7	(505/611)
Community Leader	5.7	(69/1205)	8.1	(48/594)	3.4	(21/611)
Family	6.1	(73/1205)	7.2	(43/594)	4.9	(30/611)
Friend	13.9	(168/1205)	12.1	(72/594)	15.7	(96/611)

^1 ^The denominator does not include those who declined to answer.

^2 ^For these questions, participants were asked to check all responses that apply.

### Communal Level of HIV Knowledge at Baseline

A summary of correct responses to the ten HIV knowledge questions is provided in Table 2. The overall correct response rate was between 61.9% and 83.4% for eight of the questions. The proportions of correct response were comparable between the control and intervention groups for five of the ten questions (B, C, H, I, and K). For three of the remaining five questions (A, F, and J) the proportions of correct responses were significantly higher for the control community, with knowledge higher in the intervention community for the other two (D, E). The performances of men and women were not significantly different for seven of the ten questions. However, for the remaining three questions (D, I, and K), men performed better when compared with women. Those who had no schooling performed significantly worse than those who had at least some education (Grades 1 to 6) for eight of the ten questions. For the five questions (A, D, E, F, J) for which there were significant differences in response by community group in the univariate analysis, these effects remained even after adjusting for gender and education in a multivariable logistic regression. Only knowledge related to sharing syringes (D) remained higher in men compared to women (adjusted odds ratio = 1.74, p = 0.002).

**Table 2:** HIV knowledge at baseline among 1205 participants and at 7 weeks post-intervention among 1073 participants in three rural Nigerian communities

A	Working near someone with the AIDS virus *(No)*																
B	Eating food cooked by someone who has the AIDS virus *(No)*																
C	Sharing plates, forks, or glasses with someone who has the AIDS virus *(No)*																
D	Receiving injectable drugs with syringes or needles used by someone who has the AIDS virus *(Yes)*																
E	An infant from his/her mother who has the AIDS virus *(Yes)*																
F	Being coughed or sneezed on by someone who has the AIDS virus *(No)*																
G	Having sexual intercourse with someone who has the AIDS virus *(Yes)*^1^																
H	A baby breast-fed by his/her mother who has the AIDS virus *(Yes)*																
I	Attending school with a child who has the AIDS virus *(No)*																
J	Bites by mosquitoes or other insects who have bitten a person who has the AIDS virus *(No)*																
K	Sharing a bed with someone who has the AIDS virus *(No)*																
***Baseline Correct Response Summary***																	
	**Overall** **(n = 1205)**		**Control** **(n = 594)**		**Intervention** **(n = 611)**			**Male** **(n = 643)**		**Female** **(n = 558)**			**No Education** **(n = 651)**		**Some Education** **(n = 528)**		
				
	***%***	***Total***	***%***	***Total***	***%***	***Total***	***p***	***%***	***Total***	***%***	***Total***	***p***	***%***	***Total***	***%***	***Total***	***p***
				
**A**	72.1	(836/1159)	77.0	438/569	67.5	398/590	.0003	74.9	(469/626)	68.9	(365/530)	.02	67.3	(417/620)	79.0	(406/514)	< .0001
**B**	73.2	(854/1166)	76.2	438/575	70.4	416/591	.03	74.6	(471/631)	71.4	(380/532)	.22	70.1	(438/625)	77.7	(400/515)	.0039
**C**	68.1	(789/1159)	70.0	391/559	66.3	398/600	.19	69.9	(438/627)	65.8	(349/530)	.15	64.4	(401/623)	73.4	(375/511)	.0011
**D**	83.4	(963/1155)	78.4	439/560	88.1	524/595	< .0001	88.2	(553/627)	77.6	(408/526)	< .0001	78.9	(489/620)	89.1	(457/513)	< .0001
**E**	69.5	(781/1124)	62.9	341/542	74.6	440/590	< .0001	71.6	(439/613)	65.8	(340/517)	.03	64.8	(397/613)	74.2	(368/496)	.0007
**F**	52.3	(588/1124)	57.9	309/534	47.3	279/590	.0004	51.8	(316/610)	52.9	(271/512)	.71	48.3	(291/602)	57.2	(286/500)	.0034
**H**	62.4	(708/1135)	60.4	330/546	64.2	378/589	.19	64.4	(397/616)	59.9	(309/516)	.11	61.6	(372/604)	63.2	(320/506)	.57
**I**	72.6	(817/1125)	74.5	409/549	70.8	408/576	.17	79.0	(484/613)	64.9	(331/510)	< .0001	66.4	(398/599)	79.7	(401/503)	< .0001
**J**	29.5	(337/1144)	35.6	197/554	23.7	140/590	< .0001	27.5	(171/621)	31.9	(166/520)	.11	28.9	(176/610)	30.3	(154/508)	.59
**K**	61.9	(680/1098)	63.6	333/524	60.5	347/574	.29	66.7	(396/594)	56.3	(282/501)	.0004	55.1	(325/590)	71.0	(343/483)	< .0001

1. Question G was excluded from the analysis due to an error in translation into the local Hausa language

2. Incorrect responses were people who answered "yes" or "no" inaccurately, as well as those who responded "don't know". Missing responses were excluded from these figures.

3. Bonferonni adjusted alpha (α) = 0.00

Attitudes toward HIV/AIDS were also assessed across all three communities (Table 3). Looking at the three questions which asked residents whether they agree or disagree with negative beliefs, only 25.6% of interviewees disagreed that those with HIV often are infected due to their own carelessness, while the other 74.4% either agreed or were unsure of their beliefs. There were also low disagreement rates for the latter two statements of pessimism for the prevention and mitigation of AIDS (54.5% and 39.2%, respectively). The two most common methods reported for personal protection from HIV and other sexually transmitted diseases (STDs) were abstinence and monogamy (48.0% and 38.3%, respectively). Regarding whom the participants would inform about their HIV test results, the greatest proportion of residents would tell their healthcare provider (80.9%), followed by their spouse (74.5%). People felt least comfortable sharing this information with their friends and community leaders (43.6% and 38.9%, respectively). Attitudes and beliefs were very similar between the intervention and control communities (data not shown).

**Table 3:** Attitudes and beliefs about HIV/AIDS and risk reduction among all residents of three rural Nigerian Communities (n = 1205)

	Control		Intervention	
	*(n = 530)*		*(n = 543)*	
*Question*	*%*	*Total*^1^	*%*	*Total*^1^
**The following questions ask about your beliefs regarding AIDS. Do you agree or disagree with each of the following statements?**	**Disagree**			
People who have the AIDS virus often have become infected because of their own carelessness	24.2	(116/479)	44.0	(231/525)
There is little a person can do to prevent catching the AIDS virus	54.8	(262/478)	65.3	(342/604)
Healthwise, there isn't much you can do for yourself once you have the AIDS virus	43.3	(201/464)	53.6	(281/524)

**In the past month, have you done any of the following to protect yourself from HIV and other STDs?**	**Affirmative**			
Abstained from sex	49.1	(260/530)	59.1	(321/543)
Changed the way sexual partners were selected	42.1	(223/530)	35.5	(193/543)
Reduced or limited the number of different sexual partners	42.1	(223/530)	36.6	(199/543)
Discussed HIV with sexual partner(s)	36.6	(194/530)	49.0	(266/543)
Had sex with only one partner	53.8	(285/530)	58.4	(317/543)

**If you test positive for HIV, would you tell any of the following individuals about your HIV test result?**	**Affirmative**			
Spouse	39.4	(209/530)	92.3	(501/543)
Children	37.2	(197/530)	89.3	(485/543)
Other relatives	27.9	(148/530)	85.1	(462/543)
Friends	20.4	(108/530)	81.6	(443/543)
Religious leaders	27.9	(148/530)	37.0	(201/543)
Community leaders	32.5	(172/530)	69.4	(377/543)
Physician	72.3	(383/530)	91.7	(498/543)

### Change in HIV Awareness and Knowledge

As results were qualitatively similar at weeks 7 and 14, they are only described for the first of these two follow-up visits. As shown in Tables 2 and 4, in all ten questions, participants in the intervention communities exhibited a significant net gain (all p < 0.002) in knowledge, compared with their baseline responses. This is best indicated by a far larger number of participants who answered each question correctly at follow-up after answering incorrectly at baseline, rather than the reverse change. For example, on Question A, the control group did not exhibit learning at week 7, compared to the baseline level of knowledge (p = 0.57). The net knowledge gain was significantly greater for the intervention group compared with the conventional program (p < 0.001). Looking at the whole group, 95% (488/511) of the intervention program participants answered this question correctly at the week 7 follow-up visit after 67% (342/511) provided correct responses at baseline. For the control group, the correct response rate was 78% (369/474) at week 7 and 76% (362/474) at baseline. This same pattern was observed for question B, C, F, H, I, and K with notable exceptions as follow. Regarding vertical transmission of the virus (Question E), both the control and intervention communities displayed learning at 7 weeks, with the control community performing slightly better (p < 0.0001, p = 0.0015, respectively). No difference was detected between the learning patterns of the two communities (p = 0.36). Eighty-three percent (419/505) of the intervention program participants answered this question correctly at the 7-week visit and 75% (377/505) provided correct responses at baseline. In the control community, 72% (319/446) identified the correct answer at follow-up and 59% (263/446) did so at baseline.

**Table 4:** Improved knowledge about HIV/AIDS transmission from baseline to 7 weeks post-intervention among 1073 participants in three rural Nigerian communities

Based on what you have heard about AIDS, do you think that a person can get AIDS or the AIDS virus from each of the following?							
**A. Working near someone with the AIDS virus **- *p < .0001 ^a^*							
	
**Control**				**Intervention**			
	
Baseline	Post-Intervention		**Total**	Baseline	Post-Intervention		**Total**
					
	Correct	Incorrect			Correct	Incorrect	
	
Correct	291	**71**	362	Correct	333	**9**	342
	
Incorrect	**78**	34	112	Incorrect	**155**	14	169
	
**Total**	369	105	474	**Total**	488	23	511
	
*p = .57^b^*				*p < .0001*			

**B. Eating food cooked by someone who has the AIDS virus**. - *p < .0001*							
	
**Control**				**Intervention**			
	
Baseline	Post-Intervention		**Total**	Baseline	Post-Intervention		**Total**
					
	Correct	Incorrect			Correct	Incorrect	
	
Correct	286	**73**	359	Correct	352	**3**	355
	
Incorrect	**71**	48	119	Incorrect	**145**	10	155
	
**Total**	357	121	478	**Total**	497	13	510
	
*p = .87*				*p < .0001*			

**C. Sharing plates, forks, or glasses with someone who has the AIDS virus**. - *p < .0001*							
	
**Control**				**Intervention**			
	
Baseline	Post-Intervention		**Total**	Baseline	Post-Intervention		**Total**
					
	Correct	Incorrect			Correct	Incorrect	
	
Correct	251	**72**	323	Correct	332	**6**	338
	
Incorrect	**79**	62	141	Incorrect	**167**	14	181
	
**Total**	330	134	464	**Total**	499	20	519
	
*p = .57*				*p < .0001*			

**D. Receiving injectable drugs with syringes or needles used by someone who has the AIDS virus**. - *p = .10*							
	
**Control**				**Intervention**			
	
Baseline	Post-Intervention		**Total**	Baseline	Post-Intervention		**Total**
					
	Correct	Incorrect			Correct	Incorrect	
	
Correct	312	**43**	355	Correct	428	**23**	451
	
Incorrect	**61**	45	106	Incorrect	**55**	7	62
	
**Total**	373	88	461	**Total**	483	30	513
	
*p = .08*				*p = .0003*			

**E. An infant from his/her mother who has the AIDS virus**. - *p = .36*							
	
**Control**				**Intervention**			
	
Baseline	Post-Intervention		**Total**	Baseline	Post-Intervention		**Total**
					
	Correct	Incorrect			Correct	Incorrect	
	
Correct	209	**55**	264	Correct	310	**67**	377
	
Incorrect	**110**	72	182	Incorrect	**109**	19	128
	
**Total**	319	127	446	**Total**	419	86	505
	
*p < .0001*				*p = .0015*			

**F. Being coughed or sneezed on by someone who has the AIDS virus**. - *p < .0001*							
	
**Control**				**Intervention**			
	
Baseline	Post-Intervention		**Total**	Baseline	Post-Intervention		**Total**
					
	Correct	Incorrect			Correct	Incorrect	
	
Correct	144	**107**	251	Correct	227	**8**	235
	
Incorrect	**92**	92	184	Incorrect	**233**	39	272
	
**Total**	236	199	435	**Total**	460	47	507
	
*p = .29*				*p < .0001*			

**H. A baby breast-fed by his/her mother who has the AIDS virus**. - *p < .0001*							
	
**Control**				**Intervention**			
	
Baseline	Post-Intervention		**Total**	Baseline	Post-Intervention		**Total**
					
	Correct	Incorrect			Correct	Incorrect	
	
Correct	184	**74**	258	Correct	277	**43**	320
	
Incorrect	**86**	104	190	Incorrect	**153**	35	188
	
**Total**	270	178	448	**Total**	430	78	508
	
*p = .34*				*p < .0001*			

**I. Attending school with a child who has the AIDS virus**. - *p < .0001*							
	
**Control**				**Intervention**			
	
Baseline	Post-Intervention		**Total**	Baseline	Post-Intervention		**Total**
					
	Correct	Incorrect			Correct	Incorrect	
	
Correct	264	**68**	332	Correct	337	**8**	345
	
Incorrect	**80**	34	114	Incorrect	**133**	17	150
	
**Total**	344	102	446	**Total**	470	25	495
	
*p = .32*				*p < .0001*			

**J. Bites by mosquitoes or other insects who have bitten a person who has the AIDS virus**. - *p < .0001*							
	
**Control**				**Intervention**			
	
Baseline	Post-Intervention		**Total**	Baseline	Post-Intervention		**Total**
					
	Correct	Incorrect			Correct	Incorrect	
	
Correct	86	**76**	162	Correct	99	**18**	117
	
Incorrect	**106**	184	290	Incorrect	**280**	111	391
	
**Total**	192	260	452	**Total**	379	129	508
	
*p = .03*				*p < .0001*			

**K. Sharing a bed with someone who has the AIDS virus**. - *p < .0001*							
	
**Control**				**Intervention**			
	
Baseline	Post-Intervention		**Total**	Baseline	Post-Intervention		**Total**
					
	Correct	Incorrect			Correct	Incorrect	
Correct	176	**97**	273	Correct	265	**32**	297
	
Incorrect	**71**	81	152	Incorrect	**166**	31	197
	
**Total**	247	178	425	**Total**	431	63	494
	
*p = .04*				*p < .0001*			

a. χ^2 ^test of independence, comparing changes in responses between intervention groups

b. McNemar's Test for paired responses

When gender and education were taken into account through statistical modeling, the above results did not change qualitatively. For Questions A, B, C, D, E, F, I and K, the proportions of getting the correct answer were significantly higher among those who had some education than those who had no education (all p < 0.05). For Questions D, E, G, H, I, J and K, the proportion of males getting the correct answer were significantly higher than those for females (all p < 0.05). Our assessment tool also included questions relating to attitudes and belief towards HIV/AIDS, which revealed similar responses at baseline. For the majority of these questions at the 7-week follow-up, responses improved in the intervention communities while they remained similar or declined in the control communities (data not shown). The convenience poll of the intervention communities revealed that 94.1% (447/475) of villagers interviewed had listened to the audio program.

## Discussion

In the current report, the existing level of HIV knowledge and attitudes was assessed and the effectiveness of two educational approaches in improving the level of HIV awareness was prospectively compared. Our baseline data suggested the existence of ample room for basic HIV knowledge improvement in all three villages. For a majority of the questions (6 out of 10), the correct response rates at baseline were less than 70%. Less than a third of the respondents provided the appropriate response to the question regarding the mode of HIV transmission. Factors associated with lower HIV awareness in our sample were gender, lack of formal education, and the community. These findings are important in part because women represent the fastest growing segment of the AIDS epidemic in many regions of the world, particularly in sub-Saharan Africa where over half of the disease burden is borne by females [12].

The results of our longitudinal assessment indicate that the implementation of an integrated audio program that accommodates the local learning culture is feasible and effective in enhancing HIV awareness in the rural setting. Appreciable and statistically significance changes in HIV knowledge were observed from baseline to week 7 following the integrated campaign compared with the control program of seminar/pamphlet distribution. Although the conventional approach of seminars and pamphlet distribution resulted in some amount of improved HIV awareness, the audio-device assisted intervention was associated with a more robust gain in knowledge for all but two (D and E) of the ten HIV knowledge questions. We attribute this gain to the multi-faceted approach employed in this intervention; the content was formatted into culturally-familiar oral modules and was delivered in a small group setting in the local language, by an easy-to-use audio platform. The use of an mp3-like device ensured that information was consistent and participants could refresh their knowledge by listening multiple times. Pamphlet distribution and large group seminars are widely used in HIV education in resource-limited settings, due to ease and their low cost. However, our data suggests that this approach was suboptimal compared to our novel audio approach. In the large group settings of group seminars, individuals tend to be less inclined to ask questions to clarify doubts. Furthermore, the impact of the pamphlets in rural settings is limited by low literacy. Pamphlets read during a visit by individuals unknown to the community are also unlikely to stimulate sustained discussion of the information.

Additionally, it was encouraging to find that the improvement in the level of HIV knowledge was sustained through the second assessment at week 14. Yet we had anticipated a greater gain in knowledge for the intervention group at week 14, as the delivery audio devices were left behind for subjects to replay and potentially reinforce learned messages. A number of factors may have accounted for this plateau, including learning saturation and fatigue from listening to the same content repeatedly, and inadequate time between assessment periods. Future work could include memory chips with multiple versions of the message, with variations of the songs, proverbs, parables and dramas, which could help to reduce fatigue.

Being male and having some education posed some advantage in this conservative Muslim society. Yet, regardless of subjects' gender or education status, improvements in HIV knowledge were observed across all segments of the population, supporting the value of this integrated approach as an effective mode of delivering information across demographics in these communities. The poll of audio program reach in the intervention villages indicated that device listening had proliferated in the community far beyond the original cohort of study participants. Admittedly, the convenience sampling method was suboptimal and future studies might prospectively plan to quantify program exposure throughout the community. The cost-effectiveness of deploying the digital audio outreach program in the intervention communities is noteworthy. The cost per individual educated by this intervention (including content development, technology, training and distribution), was less than US $3. This is extremely modest when compared to the cost person reached of $67 reported for HIV outreach program to prostitutes in Cameroon [13], and the estimated cost of TV and radio emission of $2566 and $383 per minute respectively [14].

Our study was limited by a number of factors including selection bias from the non-randomized selection of the communities, districts and individual participants. The follow-up of 14 weeks was rather short to assess long-term retention of HIV knowledge. Other designs (cluster trials) and sampling plans with longer follow-up should be considered when planning future studies of education outreach programs in this setting. Furthermore, the use of the same assessment instrument for all three visits may have altered patients' responses at follow-up. Future studies should consider incorporating a larger pool of questions, with several questions testing similar domains of knowledge. An assessment of the communal reach of the seminar and pamphlet program in the control village would have provided for a more robust comparison of the two intervention groups. Behavioral change resulting from knowledge gained was not directly measured; subsequent work might assess knowledge retention vis-à-vis behavior modification at later time points after program exposure.

## Conclusion

In conclusion, the low baseline HIV knowledge observed in this study the underscores the need for HIV awareness programs tailored towards the information need of these rural communities. The culturally adapted, audio HIV program that we tested offers a feasible and cost-effective alternative to improving HIV knowledge in such settings.

## Competing interests

The authors are not conflicted with regard to this study. However, Drs Ofotokun and Lennox are clinical investigators whose antiretroviral drug research projects have been funded by Abbott Laboratories, Merck Inc, and Tibotec. Dr. Kane is a founding member of ZVOX, the company which produced and supplied the audio devices used in this project.

## Authors' contributions

All authors have read and approved the final version of this manuscript.

IO, JNGB, MK, RI and, RFL, and KAE contributed to the conception and study design, protocol development, data collection, interpretation of results, writing and review of draft manuscripts.

ESR, JNGB and KAE contributed to the data management, data analysis, interpretation of results, writing and review of draft manuscripts

## Pre-publication history

The pre-publication history for this paper can be accessed here:

http://www.biomedcentral.com/1472-698X/10/2/prepub
